# Efficacy of Percutaneous Image-Guided Rupture of Lumbar Facet Cysts: A Retrospective Study

**DOI:** 10.1155/2023/5591496

**Published:** 2023-03-13

**Authors:** Yohei Ishihara, Masutaro Morishita, Koji Kanzaki

**Affiliations:** ^1^Asao General Hospital Spine Center, 6-25-1, Kamiasao, Asao-ku, Kawasaki, Kanagawa 215-0021, Japan; ^2^Department of Orthopedic Surgery, Showa University Fujigaoka Hospital, 1-30, Fujigaoka, Aoba-ku, Yokohama, Kanagawa 227-8501, Japan

## Abstract

**Background:**

Percutaneous rupture of lumbar facet cysts (LFC) is the only nonsurgical treatment which is effective in directly reducing cysts. However, this is not yet a common procedure, and its effectiveness, including the associated complications, remains unclear. Therefore, this study aimed to evaluate the clinical outcomes of percutaneous rupture for LFC and elucidate whether this minimally invasive procedure could become an alternative to surgeries for cases resistant to conservative treatments.

**Methods:**

This study investigated 57 symptomatic patients with LFC for whom conservative treatments were ineffective and underwent percutaneous rupture of the LFC. All patients were followed up for >2 years posttreatment. Clinical evaluations (visual analogue scale (VAS) and recovery rate calculated using the Japanese Orthopedic Association (JOA) scores) and radiographic evaluations (size of LFC based on magnetic resonance imaging (MRI)) were performed from pretreatment to the final follow-up examination.

**Results:**

Successful LFC rupture, without hospitalization and general anesthesia, was achieved in 48 patients. No severe complications occurred during treatment through the last observation. Satisfactory clinical results with significant improvements in the VAS and JOA scores were obtained (VAS: pre/posttreatment: 80.7 mm/11.2 mm, JOA: pre/posttreatment: 15.6 points/26.7 points, and recovery rate: 82.3%). A significant reduction in the LFC was also observed in all cases based on the posttreatment MRI findings. No successful rupture cases required subsequent surgical treatments, although four cases of LFC recurrence required additional percutaneous rupture treatment.

**Conclusions:**

Percutaneous rupture for LFC is not only a safe and minimally invasive procedure without any severe complications or requirements for hospitalization and general anesthesia but also a beneficial procedure that can eliminate the need for surgery in cases resistant to conservative treatments.

## 1. Introduction

Intracanal cystic lesions that develop adjacent to the lumbar facet joint, first described in 1968 [1], can be distinguished histologically as synovial cysts with xanthochromic fluid-filled synovial linings and ganglion cysts covered by a fibrocartilaginous capsule filled with a proteinaceous and gelatinous material excluding the synovial lining cells [2, 3]. Since both cystic lesion types are developed through indistinct degenerative changes in the facet joints, they were collectively named “facet cysts” in 1995 by Hsu et al. [4].

With the advancements in radiographic techniques, particularly magnetic resonance imaging (MRI), lumbar facet cysts (LFC) have been recognized as a common disorder, which causes severe clinical symptoms, such as lower back and leg pain because of the direct nerve compression or canal stenosis that accompanies LFC.

Janssen et al. [5] investigated the prevalence of incidental and symptomatic LFC in 19,010 consecutive patients who underwent lumbar spine MRI and found LFC in 6.5% (1228/19010) of the patients; this result was strongly associated with aging. Additionally, 54% of LFC cases identified using MRI had accompanying radiculopathy symptoms, and a large cyst size and anterior cyst location were associated with an increased likelihood of neurological symptoms.

Initial nonsurgical treatments for LFC include pain medications, bracing, physical therapy, and steroid injections. However, some previous studies have reported unsatisfactory clinical results, including unchanged cyst sizes, after these conservative treatments [4, 6]. For cases resistant to conservative treatments, surgeries were generally performed. Many reports have shown the effectiveness of surgery, with detailed evaluations of the different surgical procedures, including cyst resections with or without spinal fusion and microendoscopic decompression for cases resistant to conservative treatment [7–9]. However, surgical treatments burden patients by requiring longer hospital stays and general anesthesia induction. Additionally, previous studies have reported a higher rate of incidental durotomy during cyst resection surgery than in other degenerative lumbar disorders because of the significant changes in adhesions between the cyst and dura [10]. Percutaneous LFC rupture is a treatment option that can directly reduce the cyst size without requiring hospitalization and general anesthesia. First described in 2001, studies have reported the usefulness of this procedure, including eliminating the need for surgical interventions [11, 12].

However, this is not yet a common procedure, particularly in our country, with only a few case reports [13], and its effectiveness, including the associated complications, has not been clarified. Therefore, this study aimed to thoroughly evaluate the clinical results of percutaneous LFC rupture and elucidate whether this minimally invasive procedure could become an alternative to surgery for cases resistant to conservative treatments.

## 2. Materials and Methods

This was a retrospective study approved by the Institutional Review Board of Asao General Hospital; informed consent was obtained from all patients.

### 2.1. Patients

From 2006 to 2020, 104 symptomatic patients with LFC were in our hospital, of whom were diagnosed with LFC based on MRI findings. However, neurological symptoms were observed only on the LFC side in all 104 cases, and those where severe canal stenosis was combined with LFC or where clinical symptoms were observed on the non-LFC side or both sides were excluded from this study.

First, conservative treatments, including medications, braces, and physical therapy, were performed for all cases except six that required surgical treatment (i.e., microendoscopic resection) immediately after the diagnosis because of severe pain and leg paresthesia. Moreover, percutaneous LFC rupture was performed for resisted cases after conservative treatments.

After conservative treatments, the symptoms were gradually relieved in 41 of 98 cases (41.8%), and these 41 cases were defined as the control group.

**Figure 1:**
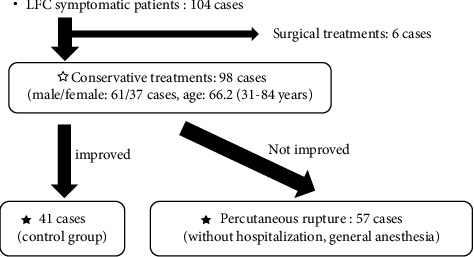
Flowchart of the treatment process for LFC. LFC, lumbar facet cysts.

Finally, we investigated 57 cases that were resistant to conservative treatments for which “percutaneous rupture of LFC” treatment was performed (Figure 1), and all patients were followed up for >2 years posttreatment (mean, 26.0 months; range, 24–40.0 months).

### 2.2. Clinical and Radiographic Evaluation

This study investigated the patient's age, sex, history of previous lumbar surgery, level of LFC, rate of successful LFC rupture, the volume of contrast agent required for successful rupture, clinical results, and intraprocedural and posttreatment complications. Clinical results were evaluated using the visual analogue scale (VAS) scores and the recovery rate, which was calculated with the Japanese Orthopedic Association (JOA) scores [9], from pretreatment to the last observation. The radiographical analysis included the presence of spondylolisthesis [14] and the grade of facet degeneration [15] at the LFC level. Lumbar lordosis (L1-S1) and the intervertebral angle on the LFC level were also evaluated from pretreatment to the last observation. The pre- and posttreatment LFC sizes were evaluated by measuring the maximum area on the MRI T2 axial slice (Mix Jam Webviewer, EBM Healthcare, Inc. Tokyo, Japan) (Table 1).

### 2.3. Statistical Analysis

Measurement values are presented as means ± standard deviations. Comparisons of clinical results and radiographical findings between the control group and the group of cases with percutaneous LFC rupture were evaluated using the chi-square test and Mann–Whitney *U* test. Additionally, comparisons of pre- and posttreatment clinical results and radiographical findings were evaluated using paired *t*-tests. A *P* value <0.05 was considered statistically significant, and all analyses were performed using JMP 13 software (SAS Institute Inc., Cary, NC, USA).

### 2.4. LFC Rupture Procedure

**Figure 2:**
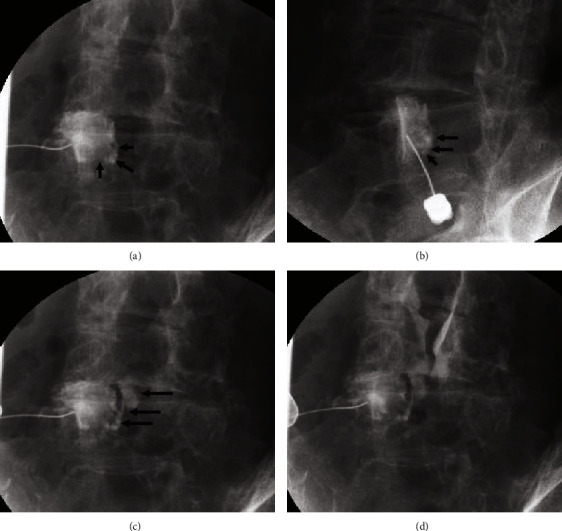
Fluoroscopic image of LFC rupture. (a), (b) Contrast imaging of the facet joint and cyst (arrow), (c) rupture of the cyst with leaked contrast agent (arrow), and (d) spread of contrast agent into the epidural space.

All procedures were performed with the patient awake, with careful attention to the patient's condition and complaints. The patients were placed in the prone position, and the right-left and cranial-caudal C-arm positions were adjusted to obtain clear views of the facet joint space. After sufficient skin disinfection, 5 mL of 1% mepivacaine (Nissin Pharmaceutical Co., Ltd., Yamagata, Japan) was administered to achieve local anesthesia. Fluoroscopic images illustrating the LFC rupture procedure from this point onward are presented in Figure 2 [16]. A 22G needle (Hakko Co., Ltd, Tokyo, Japan) was inserted into the facet joint space under fluoroscopy, and the contrast agent (Omnipaque 240, GE Healthcare Japan Corporation, Tokyo, Japan) was injected into the joint space with monitoring. After the contrast of the joint space was confirmed, an additional contrast agent was added until the LFC was visible. While carefully monitoring the patient's condition, particularly for back and leg pain complaints, an additional contrast agent was added under pressure until the LFC ruptured. LFC rupture was confirmed by the sudden feeling of a loss of resistance, concurrent with the leakage of the contrast agent into the epidural space under fluoroscopy. After the LFC rupture, 8 mL fluid, comprising 5 mL saline (VTRS, Viatris, Tokyo, Japan) and 3 mL local anesthesia, was added while monitoring the contrast agent spread into the epidural space.

After the treatment, patients were transferred to the recovery room and observed for 2 hours, monitoring their general condition, leg pain, and paralysis. All patients left the hospital after 2 hours of observation, based on the physician's assessment that no severe complications or complaints had occurred.

## 3. Results

Of the 57 cases where LFC rupture was performed, 21 (36.8%) had a history of previous lumbar surgery. The levels of LFC were L2/3, L3/4, L4/5, and L5/S in 2, 9, 36, and 10 cases, respectively. The facet degenerative changes were grades 1, 2, 3, and 4 in 5, 22, 25, and 5 cases, respectively, and degenerative spondylolisthesis was observed in 57.9% (33/57) of the cases. The grade of degenerative change and spondylolisthesis showed no significant progression by the time of the last observation posttreatment. Moreover, lumbar lordosis and the intervertebral angle on the LFC level did not change significantly until the last observation.

All patients complained of leg pain, and a slight leg weakness (manual muscle testing; 4-5) was recognized in 21.1% (12/57) of the cases. Furthermore, significant differences in the VAS and JOA scores, size of the LFC based on MRI, were recognized after comparison with the corresponding scores of the control group (*P* < 0.01).

**Figure 3:**
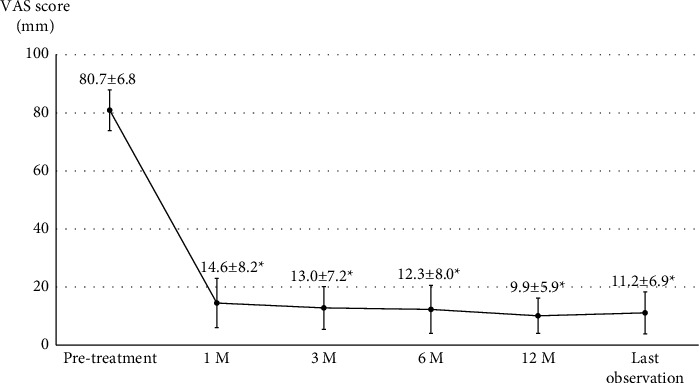
VAS scores from pretreatment to last observation. M, months after treatments; VAS, visual analogue scale. ^*∗*^Significant difference compared with pretreatment (*P* < 0.0001).

**Figure 4:**
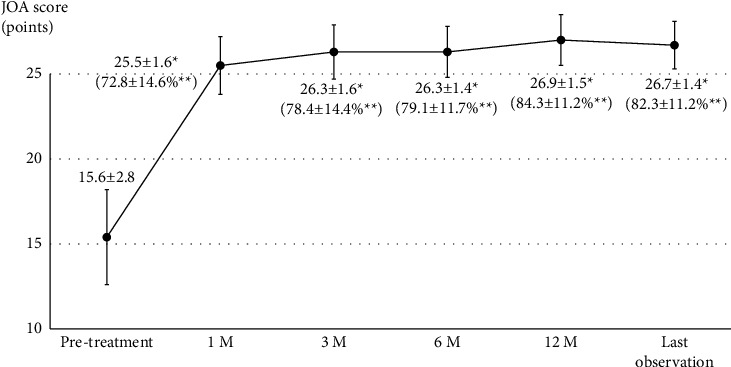
JOA scores from pretreatment to last observation. M, months after treatments; JOA, Japanese Orthopedic Association. ^*∗*^Significant difference compared with pretreatment (*P* < 0.0001); ^*∗∗*^Recovery rate.

Successful LFC rupture was achieved in 48 patients, and the average volume of contrast agent to achieve a rupture was 2.3 ± 0.4 mL. No severe complications occurred during the treatment, and all patients left the hospital after a 2-hour observation without severe complaints, including worsening pain or paralysis. Additionally, severe complications were not observed by the time of the last observation, and satisfactory clinical results were obtained, with a significant improvement in the VAS score (*P* < 0.0001, Figure 3) and JOA score with 82.3 ± 11.2% recovery rate (*P* < 0.0001, Figure 4).

Additionally, MRI evaluation was performed for 40 patients at 2–6 months after treatment, and a significant reduction of LFC was recognized in all 40 cases (pre-/posttreatment: 62.1 ± 34.7 mm^2^/12.0 ± 6.5 mm^2^, *P* < 0.0001). By the time of the last observation, no cases of postsuccessful rupture required subsequent surgical treatments; meanwhile, LFC recurrence accompanied by leg pain was observed in four cases within 1 year following the MRI's confirmation of reduction, after which the treatment was repeated. After the second injection, the cyst reduction was reconfirmed without further recurrence (up to 24 months after the second injection). In contrast, the rupture of the cyst failed in nine patients. In three patients, needle insertion was impossible because of severe degeneration of the facet joint. In the other six patients, despite successful needle insertion into the facet joint space, the cyst was not contrasted clearly, and the contrast agent leaked outside the joint. Of the nine failed rupture cases, seven underwent surgical treatment (microendoscopic resection) after 2–6 weeks. In the remaining patient, although the pain was not relieved, the patient did not agree to undergo surgery and preferred to undergo conservative treatments (Table 2).

### 3.1. Case 1 (Figure 5 [16])

Successful rupture of the cyst was achieved in an 81-year-old male patient. The pain was relieved immediately after the treatment, and cyst reduction was confirmed on MRI 3 months posttreatment. By the time of the last observation (after 2 years), the patient had no complaints. The JOA score improved from 14 points at pretreatment to 29 points at the last observation, with a 100% recovery rate.

### 3.2. Case 2 (Figure 6 [16])

In the case of a 65-year-old female patient, successful cyst rupture was not achieved, and leakage of the contrast agent outside the joint and unimproved symptoms were observed. Two weeks later, microendoscopic resection was performed. The JOA score was improved from 5 points at pretreatment to 23 points at the last observation, with a 75% recovery rate.

## 4. Discussion

LFC most commonly occurs at the L4-L5 level due to excessive loading of the facet joint involved with chronic higher mobility of the spinal segment, which is particularly found in degenerative spondylolisthesis [3–5]. Kusakabe et al. [3] radiologically and histopathologically investigated 46 cases of LFC, where the communication channel between the cyst and the facet joint was confirmed after evaluation using computed tomography arthrography. They proposed the pathogenesis of LFC formation as degenerative arthritic changes and spinal instability that first cause ligament flavum degeneration, followed by fissures in the collagen capsular portion of the ligament flavum that develop into LFC, accompanied by joint fluid from the connecting facet joint and secretions from fibroblasts. In our study, 61.2% of LFC (60/98 cases) were observed at the L4/5 level, and 52.0% (51/98 cases) showed instability with spondylolisthesis, consistent with previous reports.

Previous studies have also described the occurrence of LFC after posterior decompression surgery of the lumbar spine [17, 18], which is consistent with our results demonstrating 39 (39.8%) postoperative cases, 17 and 22 occurring after fenestration and microendoscopic decompression, respectively.

Ikuta et al. [17] found that the prevalence of postoperative LFC after decompression surgery for lumbar spinal stenosis was 8.6%. They speculated that the cause of postoperative LFC development was the weakening of the medial portion of the facet joint after ligament flavum removal and mechanical stresses on the medial portion of the joint, such as postoperative segmental spinal instability. This instability was proposed to include a progression of spondylolisthesis and disc degeneration and to cause the joint capsule on the treated side to protrude easily into the spinal canal.

Many surgical procedures for LFC were reported previously, such as cyst resection with or without spinal fixation [7–9]. Cyst resection with facetectomy and spinal fusion is the most reliable procedure for preventing cyst recurrence [7]; however, the highly invasive nature of the surgery and adjacent segmental disorders create postoperative challenges. Kusakabe et al. [8] performed en-bloc cyst resection through fenestration without fixation for 96 patients (mean postoperative follow-up period: 2.5 years) and obtained satisfactory clinical results, with a JOA recovery rate of 86% and a recurrence rate of 2.1%. Some recent studies also suggested that microendoscopic resection is the most minimally invasive surgical procedure, with a small skin incision, limited tissue dissection, and improved visualization [9]. In contrast, surgeries for LFC require careful attention regardless of the surgical procedure due to adhesions between the cyst and dura that cause dural tears [8, 10, 19]. Takahashi et al. [10] reviewed 1014 cases where surgical procedures were performed for degenerative spinal disease, including 22 LFC cases, and reported a total incidence rate of durotomy of 4% (41 cases). In contrast, the durotomy incidence rate of 18% (4/22 cases) noted among LFC cases was much higher than that observed in cases with other degenerative disorders, such as disc herniation (2.0%), stenosis (1.8%), and degenerative spondylolisthesis (9%). Additionally, as demonstrated in our study, some LFC occurred after lumbar surgeries that involved more severe adhesions and were related to a higher risk of dural tear [19]. As a primary issue, surgical treatments cannot eliminate the most severe disadvantages for patients, which include longer hospital stays and general anesthesia induction.

Besides surgical treatments, percutaneous LFC rupture was the only treatment that could directly reduce cysts without hospital stay or general anesthesia induction. The systematic investigation by Martha et al. [12] included the largest sample size (101 cases) for this ambulatory treatment technique, demonstrating successful cyst rupture in 81% of cases, similar to our 84.2% (48/57 cases) successful rupture rate. However, 54% (55/101) of patients, including those with successful ruptures, required subsequent surgery because of continued symptoms. Ιn our study, no patients with successful cyst rupture required subsequent surgery based on confirmation of cyst reduction on MRI and satisfactory pain relief without significant progression of spinal instability accompanied by the absence of significant change in spondylolisthesis, lumbar lordosis, and the intervertebral angle at the last observation. The overall rate of surgery prevention was higher in our study than that in others previously published [11, 12, 20]. The most important procedural difference between ours and previous studies was that our procedure included an additional 8 mL fluid into the facet joint after confirmation of successful cyst rupture. Although the effectiveness of adding saline and mepivacaine after the rupture had not been previously demonstrated, the cyst might be decompressed immediately via enlargement of the ruptured hole, which may help reduce the cyst and relieve the pain. Furthermore, our procedure may involve a healing process since flushing pain-related substances out of the facet joint and from around the compressed nerve root allows their passage into the epidural space. This may enable phagocytes to accumulate in a similar mechanism to that of the percutaneous intradiscal high-pressure injection of saline into lumbar herniated discs proposed by Fukui et al. [21].

No severe LFC rupture complications had been reported previously. In our study, some patients complained of low back or leg pain during the procedure, particularly when the cyst burst; however, the pain decreased after the treatment without deteriorating to lower-extremity paralysis, and all patients left the hospital after 2 hours of rest. By the time of the last follow-up examination, no severe complications were observed in any of the cases. Furthermore, since percutaneous facet resolution may lead to complications, such as intravascular penetration, infection, hematoma, and vasovagal reactions [22, 23], careful attention is essential during the procedure, and regular follow-up examinations are necessary to monitor the condition.

Our study had some limitations, particularly regarding the follow-up period and sample size. The average follow-up period in our study was 26.0 months; therefore, the long-term effectiveness, including the recurrence risk, was not elucidated. Huang et al. [24] reported the long-term outcomes of 71 cases with an average follow-up period of 44 months, where repeated cyst rupture was performed in 8 cases (12%). However, six of the eight cases eventually required surgery for LFC resection, showing that the procedural effectiveness may decrease for recurrent cases. Although the cyst reduction was recognized on MRI after treatment in our study, the risk of LFC developing because of facet joint degeneration or spondylolisthesis remained. Therefore, continuous long-term observation is necessary posttreatment, even with pain relief and cyst reduction. Although this study was the first in our country, except for case reports, to investigate the effectiveness of percutaneous cyst rupture, the sample size was still small. In our study, the cyst rupture failed in nine cases, with severe joint degeneration as the cause of failure in three cases. For the other six cases, the cause was not determined; however, the communication between the joint and cyst might have been defective. Furthermore, by continuing this treatment for more cases, the limitations of this treatment, including identifying resistant cases that should receive surgery, may be clarified in the future.

## 5. Conclusions

Our clinical results showed satisfactory effectiveness with the “percutaneous rupture for LFC” procedure since the rate of surgery prevention after this treatment was 84.2% (48/57 cases).

Percutaneous rupture for LFC is safe and minimally invasive without any severe complications, need for hospitalization, or general anesthesia. Moreover, it is a very useful procedure that can prevent surgery in cases resistant to conservative treatments. Therefore, percutaneous rupture for LFC should be the first-choice treatment in cases resistant to conservative treatment instead of surgical treatments.

## Figures and Tables

**Figure 5:**
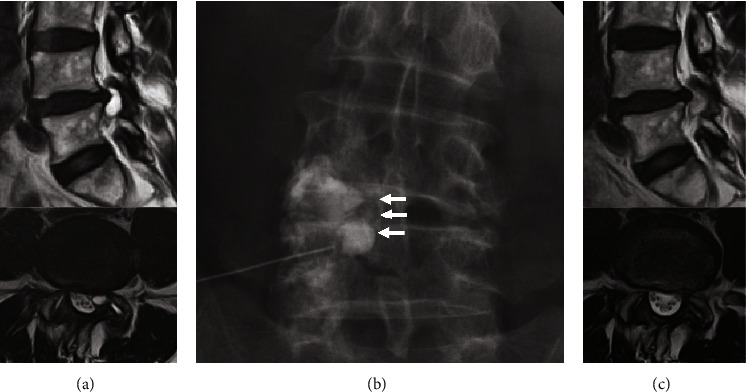
The case with successful rupture of the cyst: (a) pretreatment MRI, (b) successful rupture of the cyst (arrow: leak of contrast agent), (c) posttreatment MRI. MRI, magnetic resonance imaging.

**Figure 6:**
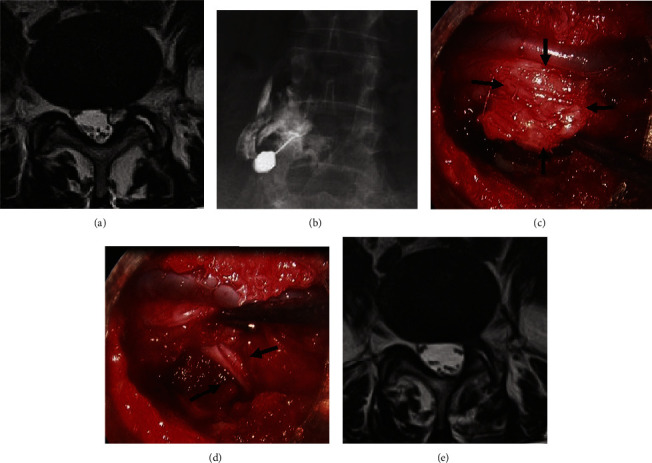
The case with failed rupture of the cyst: (a) pretreatment MRI, (b) failed rupture of the cyst, (c) before cyst resection (arrow; cyst), (d) after cyst resection (arrow: L5 root), (e) postoperative MRI. MRI, magnetic resonance imaging.

**Table 1:** Demographic data of patients undergoing cyst rupture (*N* = 57) and those included in the control group (*N* = 41).

	Cases with cyst rupture	Control group	*P* value
Sex			0.295
Male/Female	33/24 cases	28/13 cases	
Age; mean (range)	65.6 (31–82) years	67.0 (35–84) years	0.519
Lumbar level of cyst			0.783
L2/3 : L3/4 : L4/5 : L5/S	2 : 9 : 36 : 10 cases	3 : 8 : 24 : 6 cases	
History of previous lumbar surgery			0.481
No	36 cases	23 cases	
Yes	21 cases	18 cases	
Fenestration	10 cases	7 cases	
Microendoscopic decompression	11 cases	11 cases	
Grade of facet joint OA			0.868
Grades ½	5/22 cases	0/24 cases	
Grades ¾	25/5 cases	11/6 cases	
Degenerative spondylolisthesis			0.171
No	24 cases	23 cases	
Yes	33 cases	18 cases	
Grades ½	27/6 cases	16/2 cases	
Lumbar lordosis	39.6 ± 7.3°	40.7 ± 6.7°	0.582
Intervertebral angle	5.7 ± 2.6°	6.4 ± 2.5°	0.206
Area of cyst	56.9 ± 31.3 mm^2^	41.5 ± 16.3 mm^2^	**0.009** ^*∗*^
VAS score	80.7 ± 6.8 mm	59.4 ± 12.3 mm	**<0.0001** ^*∗*^
JOA score	15.6 ± 2.8 points	20.9 ± 2.9 points	**<0.0001** ^*∗*^

^*∗*^Significant difference between cases with cyst rupture and the control group (*P* < 0.01). OA, osteoarthritis; JOA, Japanese Orthopedic Association.

**Table 2:** Results of percutaneous image-guided rupture of lumbar facet cysts (*N* = 57).

Rupture of the lumbar facet cyst	
Successful rupture	48 cases (84.2%)
Failed rupture	9 cases (15.8%)
Volume of contrast for rupture^*∗*^	2.3 ± 0.4 mL (1.7–4.0 mL)
VAS score^*∗*^	
Pre-/posttreatment	80.7 ± 6.8/11.2 ± 6.9^*∗∗∗*^mm^2^
JOA score and recovery rate^*∗*^	
Pre-/posttreatment (recovery rate)	15.6 ± 2.8/26.7 ± 1.4^*∗∗∗*^points (82.3 ± 11.2%)
Area of cyst^*∗∗*^	
Pre-/posttreatment	62.1 ± 34.7/12.0 ± 6.5^*∗∗∗*^ mm^2^
Cyst recurrence after successful rupture	4 cases
Needed surgical treatment	0 case: after successful rupture
	7 cases: after failed rupture

^*∗*^Only 48 cases with successful cyst rupture. ^*∗∗*^Only 40 cases in which MRI was performed after a successful cyst rupture. ^*∗∗∗*^Significant difference between pre- and posttreatment (*P* < 0.0001). JOA, Japanese Orthopedic Association; VAS, visual analogue scale.

## Data Availability

The datasets generated and/or analyzed during the current study are not publicly available due to the data sharing policy of Asao General Hospital but are available from the corresponding author on reasonable request.
